# Barriers in utilization and provisioning of obstetric care services (OCS) in India: a mixed-methods systematic review

**DOI:** 10.1186/s12884-023-06189-x

**Published:** 2024-01-02

**Authors:** Sushmita Singh, Rahul Rajak

**Affiliations:** 1 Centre of Social Medicine and Community Health, Jawaharlal Nehru University, New Delhi, India; 2 Institute of Development Studies, Kolkata, India

**Keywords:** Obstetric care service, Barriers, Maternal Health, Systematic Review, India

## Abstract

**Background:**

Despite the reduction in the maternal mortality ratio, barriers in obstetric care services (OCS) remain a significant risk factor for adverse maternal and perinatal outcomes in India. This review covers the ‘continuum of care’ (ANC, child delivery, and PNC services) and identifies multiple barriers in provisioning as well as utilization of OCS in India. We conducted a systematic review to understand the barriers using a mixed-methods approach.

**Methods:**

PubMed, Scopus, Web of Science, Google Scholar, and Science Direct databases were searched from 1 January 2000 to 30 June 2022. The methodological quality of the included studies was assessed using appropriate tools. After a full-text review of 164 studies, total of 56 studies (33 quantitative, 18 qualitative, and 5 mixed-methods studies) were finally included in the review. All the barriers were classified into five major themes: (i) individual and interpersonal barriers, (ii) social and cultural barriers, (iii) structural barriers, (iv) logistical barriers, and (v) organizational barriers. A thematic synthesis approach was used to present the findings of the included studies.

**Results:**

Lack of knowledge and awareness and less family support in availing the required OCS were key individual and interpersonal barriers. Negative social and cultural practices, such as belief in traditional herbs/healers, dietary restrictions, and discarding colostrum were frequently reported barriers, especially in rural settings. Poor economic status and high health service costs were the most often cited barriers to low institutional delivery and delayed ANC services. Long distances to health facilities and poor road conditions were the most frequently reported logistical barriers. On the provisioning side, poor quality of treatment, shortage of drugs and equipment, and non-cooperative attitude of health professionals were the most significant barriers.

**Conclusion:**

This review identified several important barriers ranging from individual and cultural to structural, logistical, and organizational, which are prevalent in India. To mitigate the barriers, the governments need to develop strategies at the individual and organizational levels. Innovative interventions and program implementation at the community and village levels could also be contributory steps towards improving OCS utilization in India.

**Supplementary Information:**

The online version contains supplementary material available at 10.1186/s12884-023-06189-x.

## Background

The existing studies indicate that inadequacy or lack of obstetric care services (OCS) are the primary cause of maternal and perinatal complications in India, which lead to infant and maternal mortality [1–3]. The concept of OCS has three interrelated and sequential dimensions, i.e., antenatal care (ANC), care during delivery, and postnatal care (PNC). It is well-known and widely accepted that OCS significantly contributes to keeping mother and child healthy and lowering the risk of adverse maternal and perinatal outcomes [2–5]. Yet, globally, every year 0.29 million mothers lose their lives due to complications of pregnancy and childbirth [6]. India has registered a steady decline in the maternal mortality ratio (MMR) from 113 per 100,000 live births in 2016 to 103 in 2019 [7], but barriers in OCS remain significant risk factors for maternal mortality in most of the states, especially in the “BIMARU” (Bihar, Madhya Pradesh, Rajasthan, and Uttar Pradesh) states [3, 8]. Caste and class-based disparities and the urban-rural differential are the other obstacles that hinder the utilization of OCS in India [8–10].

In 2019, the World Health Organization (WHO) identified five major OCS-related barriers, namely (i) poverty, (ii) lack of information, (iii) distance to health facilities, (iv) inadequate and poor OCS, and (v) cultural beliefs and practices [6]. Likewise many previous studies in India have identified similar barriers to utilizing the ANC [2, 5, 9, 11–16], child delivery and breastfeeding [17–25], and PNC services [26–30]. It is pertinent to highlight here that according to the most recent National Family Health Survey [NFHS-5], until as recently as 2021, 49% of mothers had not received full ANC services and 38% of mothers had not delivered in a public facility [31].

In India, OCS barriers are mostly categorized in demand and the supply sides [12]. Lack of knowledge and awareness, lack of women’s autonomy, traditional and alternative practices, fear and stigma, influence of family members, long distance to health facility, financial constraints, poor quality of health service, and poor attitudes of medical staff have been consistently reported as significant demand-side barriers [4, 29, 32–35]. On the other hand, shortage of drugs, medical equipment, and human resources and lack of physical infrastructure are prominent barriers in supply side [3, 12, 25, 36]. All these barriers are aggravated among the socially and economically disadvantaged ethnic minority groups [8, 27, 34].

To date, the systematic reviews of barriers have primarily focused on specific components of OCS, with centrality accorded to either demand-side or supply-side barriers [37–39]. Only a few studies have combined both demand- and supply-side barriers to address the full range of obstacles in OCS [12, 33, 34]. This study aims to fill this research gap by synthesizing the available literature on barriers in OCS that affect different aspects of mother and child health. This review covers the continuum of care (ANC, child delivery, and PNC services) and identifies multiple barriers in utilization and provisioning of OCS in India. It covers a wide range of literature that encompasses qualitative, quantitative, and mixed-methods studies. The holistic approach of this systematic review distinguishes it from the existing systematic literature reviews.

Based on this background and relevance, the main aim of this study is to understand the barriers in utilization and provisioning of OCS in India by performing a mixed-methods systematic review. Identifying and understanding these barriers can help develop effective strategies to improve maternal health care services.

## Method

We carried out a systematic mixed-methods review for a thematic synthesis of the findings of the included studies and adopted the Preferred Reporting Items for Systematic Reviews and Meta-Analysis (PRISMA) guidelines (Additional File 1) to report the findings of the review.

### Systematic search strategy

An extensive search was undertaken from April 2022 to June 2022 for peer-reviewed articles across several electronic databases (PubMed, Scopus, Web of Science, Google Scholar, and Science Direct). The reference lists of the included studies were also reviewed for additional relevant articles. The search was performed independently by two researchers (SS and RR) by using three broad categories of keywords and MeSH descriptor terms, that is, “obstetric care services” OR “utilization of obstetric care services” OR “provisioning of obstetric care services”, along with their synonyms and closely related words. The complete list of search terms is provided in (Additional File 2). These terms were combined using Boolean operators in this format: ‘(person) AND (service) AND (utilization).’ Duplicates from the results retrieved from all the databases were identified and removed.

**Table 1:** Inclusion and exclusion criteria. The PICO (Population, Intervention [or exposure], Comparison, and Outcome) framework was used for study selection [42]

Criteria	Inclusion	Exclusion
Population	**Service user**: Women of reproductive age (15–49 years), pregnant women, postnatal women. **Family member of service user**: Husband, household head, mother-in-law **Service provider**: Health care workers (ANM, ASHA, Anganwadi worker, Medical officer), traditional birth attendants, community leaders	Women who had not received any of the components of the continuum of care (ANC, child delivery, and PNC services)
Relevance	Only peer-reviewed articles published in the English language	Commentaries, grey literature, working papers, and review articles
Time	Journal articles published between 1 January 2010 and 30 June 2022	Articles published before 1 January 2010 or after 30 June 2022
Intervention	Not restricted	Not applicable
Setting/Location	India at country, state, multi-state, city, or population group levels	Any study conducted outside of India
Study types and designs	Quantitative, qualitative, and mixed-method studies	Not fit in the quality assessment process Full text not accessible
Control/Intervention	Not restricted	Not applicable
Outcome	Studies that identified barriers to any component of continuum of care (ANC, child delivery, and PNC services) or combined studies	Studies that only examined maternal health or their components without reporting any barriers and/or factors

### Screening of the articles

The articles were screened independently by two reviewers (SS and RR), and the discrepancies resolved by consensus. In the initial screening, both of authors (SS and RR) independently screened the titles and abstracts that met the inclusion criteria. Afterward, all the relevant studies were imported into Covidence, a web-based systematic review software for remove the duplicates and screening titles and abstracts [40, 41]. This process was followed double screening for identified the potentially eligible studies. To validate the data screening process, first reviewers (SS) crosschecked the 10% (n = 104) randomly selected abstracts and read carefully and confirm whether a selected article should proceed to full-text eligibility or not. Studies that both the reviewers agreed on were included in the full-text review. Any disagreement between the reviewers was handled through discussion and consensus to minimize bias. The full-text articles were then extracted and assessed against the inclusion and exclusion criteria (Table 1). The selected full-text articles were re-evaluated for data extraction and assessed for quality.

### Quality assessment and risk of bias

To assess the quality of the included papers, we adopted separate methods for qualitative, quantitative, and mixed-methods studies. For qualitative studies, we adopted the “Critical Appraisal Skills Programme,” which contains a checklist of 10 questions [43]. Each question has three options (1) Yes (score: 1.0), (2) Partial Yes (score: 0.5), (3) No (no score). We classified studies as good, moderate, or low-quality if they scored 9–10, 7.5–8.5, or 6–7.0, respectively. For quantitative studies, the “Quality Assessment Tool for Observational Cohort and Cross-Sectional Studies” tool was used. This tool was developed by the UN National Heart, Lung, and Blood Institute [44]. This tool has 14 criteria outlined for the appraisal. Each question has three options (1) Yes (score: 1.0), (2) No (score: 0), and (3) Other (not included) [Cannot Determine: CD; Not Applicable: NA; Not Reported: NR]. Taking into consideration each criterion, the researchers evaluated the overall quality of a study as Good (11–14 score out of 14 questions), Fair (5–10 score out of 14 questions), and Poor (0–4 score out of 14 questions). The “Mixed-methods appraisal tool (MMAT) version 2018” seemed to be the best tool to assess mixed-methods studies [45]. In this tool, a set of five questions available to assess the quality of mixed-methods articles. There are three response options: ‘Yes’ (Score:1) means that the criterion is met, ‘No’ (Score: 0) means that the criterion is not met, and ‘Can’t tell’ (No Score).

### Data extraction and synthesis

Data extraction was conducted in two phases. In the first phase, data extraction forms were used to record the basic characteristics of the included studies (study methods, study focused areas [ANC, child delivery, and PNC services], study year, sample size, method of data collection, data source, and geographic setting). The authors reviewed all the included studies and extracted the key barriers from each study. Also, we classifies the barriers into five major themes, namely:

1. Individual and interpersonal barriers.
2. Social and cultural barriers.
3. Structural barriers.
4. Logistical barriers.
5. Organizational barriers.

Furthermore, we have also divided each theme into three categories, i.e., qualitative, quantitative, and mixed – method studies.

In the second phase, the authors adopted a thematic synthesis approach for all the selected studies to present the main findings [46]. For the synthesis, the findings of the included studies were categorized across five major themes. This narrative finding allowed us to capture similarities and variations across the included studies. From the qualitative studies, the authors extracted specific quotes for each thematic analysis. The quotes were concise examples of common themes found across many articles. For data validation, extracted data was rechecked in a data extraction form. Disagreements in data extraction were resolved through discussion between both the authors until a consensus was gained. A sample of the abstracts was also given to the first reviewer (SS) for validation. Sample data extraction forms are provided (Additional File 3).

## Results

### Study population

The study population was identified as service users, that is, women of reproductive age (15–49 years), pregnant women, and postnatal women, and, in a few cases, family members of service users such as husband, household head, and mother-in-law. A few studies focused on service provider (ANM, ASHA, Anganwadi worker, Medical officer), maternity care workers, traditional birth attendants, and community leaders.

### Conceptual framework

**Fig. 1:**
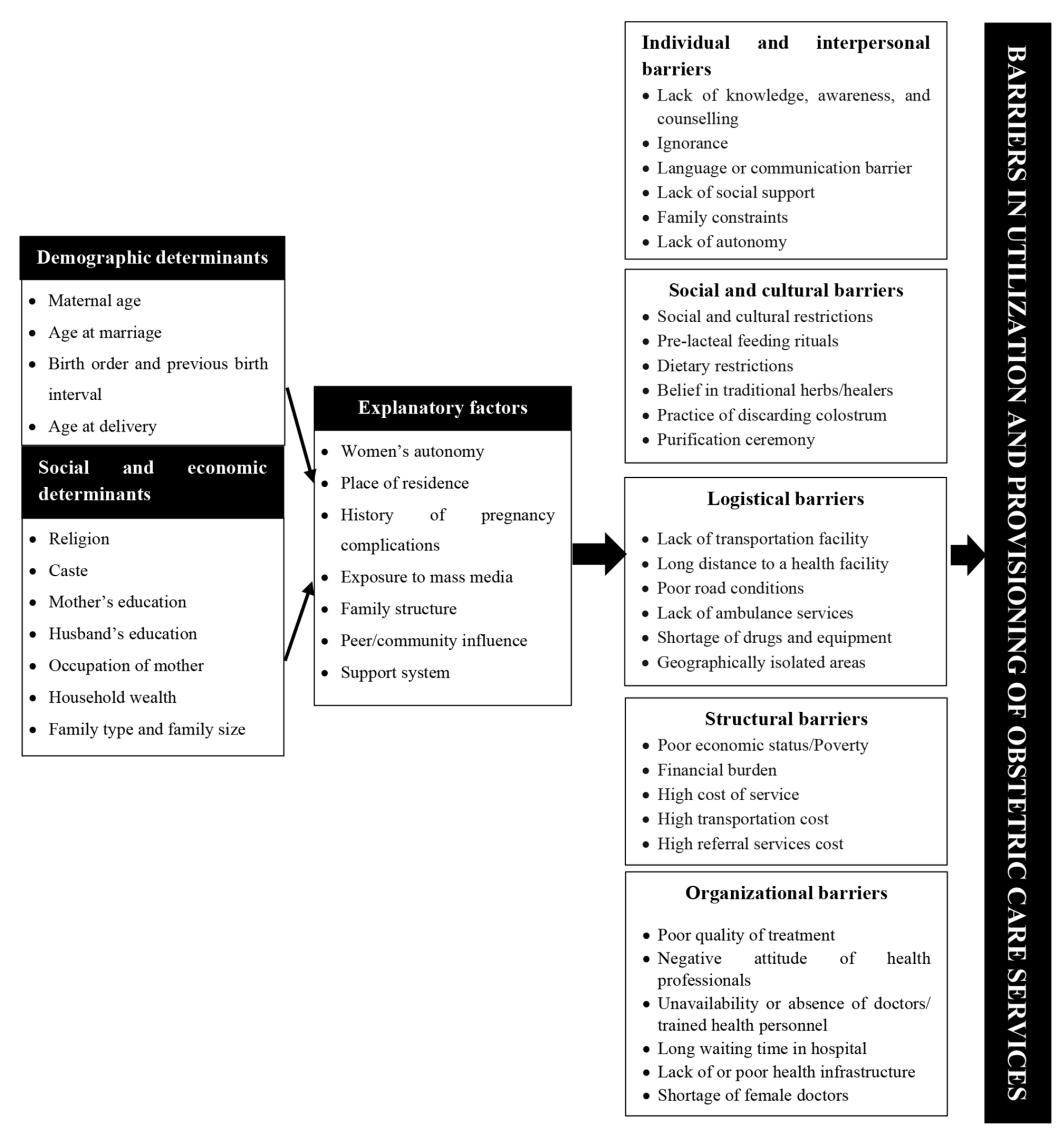
Conceptual framework of barriers in utilization and provisioning of OCS in India. This conceptual framework was developed by the authors

A conceptual framework was developed to organize the barriers in OCS and examine the associated factors. The framework includes the major demographic, social, and economic determinants that influence OCS. The framework also explored the explanatory factors which are inter-connected with the barriers. A detailed integration of the barriers is exhibited in Fig. 1.

### Selection of articles

**Fig. 2:**
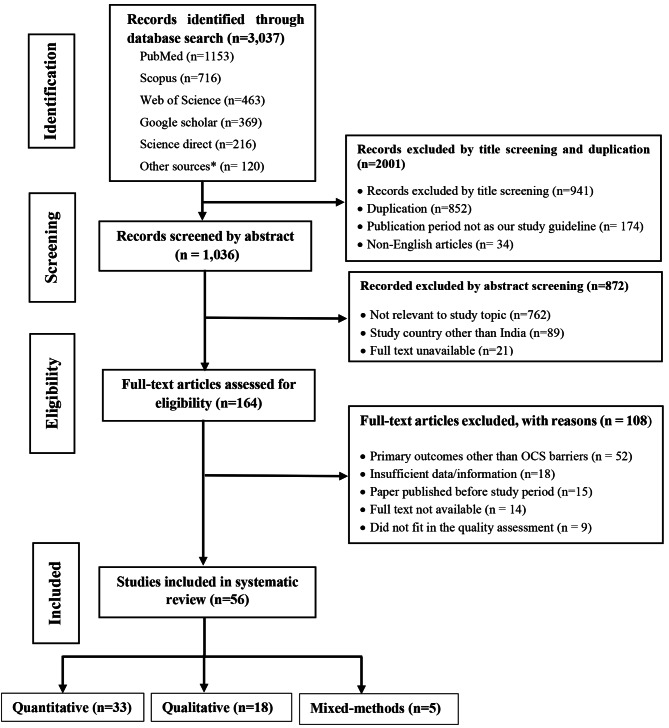
Flow chart of selection process of eligible studies **Note: Other source**: Cross-reference check of included studies

A total of 3,037 studies were initially identified through a search performed on five different databases using different keywords. Out of them, 2001 studies were removed based on title screening and duplication. After screening the articles by their abstract, 872 were found to not meet the inclusion criteria. The remaining 164 full-text articles were further assessed for eligibility. Out of them, 108 were excluded based on multiple reasons such as primary outcomes other than OCS barriers (n = 52), insufficient data/information (n = 18), and lack of fit as per quality assessment (n = 9). Finally, 56 studies were included in the review. The study selection flow diagram is presented in (Fig. 2).

### Methodological quality of studies

All the studies included in the review were rated as being good-to-low in quality. However, out of 18 qualitative studies, 7 were assessed as low quality (score between 6.0 and 7.0). Among the quantitative studies, all the included studies were rated as being in the good-to-moderate quality category. Out of the 5 mixed-methods studies, 4 were rated as good (score 4–5 out of 5 questions). Overall, all the included studies lacked bias and were found to be satisfactory in quality assessment. The full explanation of the quality assessment of all the three types of studies is given in Additional File 4.

### Characteristics of the included studies

The descriptive statistics of the study sample show that a total of 56 studies were included in the systematic review. About 59% (n = 33) of the included studies employed a quantitative design, 32% (n = 18) were qualitative in nature, and 9% (n = 5) were mixed-methods studies. Of all the included studies, 28.6% (n = 16) focused on antenatal care, 21.4% (n = 12) focused on breastfeeding, and 16.1% (n = 9) covered the whole continuum of care. The majority of the studies were published during the period 2019–2021 [(2019 year: 14.3%, n = 8,), (2020 year: 9.6%, n = 11), (2021 year: 12.5%, n = 7)]. The most of the studies, 82% (n = 46) used primary data collection. In terms of data collection method, 32% (n = 18) of the included studies were community-based cross-sectional studies and 25% (n = 14) studies focuses on Key informants/IDI and FGD/Case study. The full explanation of the characteristics of the included studies is well illustrated in Table 2.

**Table 2:** Characteristics of included studies (n = 56)

General characteristics of studies	N (%)	Study Reference
**Study methods**		
Quantitative	33(58.9)	[2–5, 8, 9, 11–22, 24, 28–30, 32, 33, 36, 47–51, 61, 65, 66]
Qualitative	18 (32.1)	[1, 23, 26, 27, 34, 35, 52–56, 59, 62–64, 67–69]
Mixed-Methods (qualitative + quantitative)	5 (8.9)	[10, 25, 57, 58, 60]
**Study’s focus**		
Antenatal care	16 (28.6)	[2, 3, 5, 8, 9, 11–16, 48, 54–56, 59]
Breastfeeding	12 (21.4)	[18–24, 26, 49, 52, 53, 57]
Delivery (institutional/home)	5 (8.9)	[17, 25, 64, 68, 69]
Postnatal care	8 (14.3)	[1, 27–30, 61, 62, 67]
Antenatal care and delivery care	2 (3.6)	[4, 34]
Antenatal care and postnatal care	3 (5.4)	[32, 47, 63]
Postnatal care and delivery care	1 (1.8)	[10]
Continuum of care (ANC, child delivery, breastfeeding, PNC)	9 (16.1)	[35, 36, 50, 51, 58, 60, 65–67]
**Study year**		
2010	1 (1.8)	[17]
2011	3 (5.4)	[4, 32, 51]
2012	-	-
2013	2 (3.6)	[33, 50]
2014	5 (8.9)	[18, 29, 30, 36, 58]
2015	2 (3.6)	[9, 68]
2016	5 (8.9)	[11, 12, 25, 53, 63]
2017	4 (7.1)	[47, 55, 60, 64]
2018	6 (10.7)	[1, 27, 34, 61, 67, 69]
2019	8 (14.3)	[2, 5, 19–21, 28, 54, 66]
2020	11 (19.6)	[13, 14, 22, 23, 26, 48, 49, 52, 56, 59, 62]
2021	7 (12.5)	[8, 10, 15, 16, 24, 57, 65]
2022	2 (3.6)	[3, 35]
**Data source**		
Primary data	46 (82.1)	[1, 4, 9–11, 13–16, 18–27, 29, 30, 33–35, 47, 49–64]
Secondary data	10 (17.9)	[2, 3, 5, 8, 12, 17, 28, 32, 36, 65]
**Sample size**		
< 50	10 (17.9)	[1, 26, 33, 35, 52, 53, 54, 55, 56, 68]
50–100	7 (12.5)	[10, 22, 23, 27, 34, 58, 59]
101–200	8 (14.3)	[15, 16, 49, 50, 61, 62, 64, 69]
201–500	13 (23.2)	[4, 9, 11, 13, 19–21, 24, 25, 30, 47, 57, 63]
501–1000	5 (8.9)	[14, 18, 29, 33, 51]
More than 1000	13 (23.2)	[2, 3, 5, 8, 12, 17, 28, 32, 36, 65, 48, 60, 66]
**Method of data collection**		
NFHS/DLHS/CNSS	9 (16.1)	[2, 3, 8, 12, 17, 28, 32, 36, 65]
Community-based cross-sectional Study	18 (32.1)	[4, 5, 9, 11, 13–15, 20, 21, 24, 29, 30, 33, 46, 49–51, 65]
Cohort study	1 (1.8)	[57]
Hospital/clinic based cross sectional study	6 (10.7)	[16, 18, 19, 22, 49, 61]
Key informants/IDI	7 (12.5)	[1, 26, 34, 35, 55, 59, 64]
FGD/Case study	7 (12.5)	[23, 27, 53, 54, 62, 63, 68]
FGD and IDI	4 (7.1)	[67, 52, 56, 69]
Community-based cross-sectional study and FGD/IDI	4 (7.1)	[10, 25, 58, 60]
**Geographic settings**		
National ^a^	6 (10.7)	[2, 12, 17, 36, 64, 65]
Multistate ^b^	1 (1.8)	[5]
EAG focused states ^c^	2 (3.6)	[3, 8]
State ^d^	5 (8.9)	[1, 23, 28, 32, 63]
District/Sub-district level ^e^	16 (28.6)	[14, 15, 20, 21, 23, 24, 29, 33–35, 48, 60, 62, 66–68]
Other ^f^	26 (46.4)	[4, 9–11, 13, 16, 19, 22, 25–27, 30, 47, 49–59, 61, 69]

**Note: N.A**: Not available; **NFHS**: National Family Health Survey; **DLHS**: District Level Health Survey; **CNSG**: Comprehensive Nutrition Survey in Gujarat; **FGD**: Focus group discussion; **IDI**: In-depth interview

^**a**^ Includes national data for India; includes studies conducted in India at country level

^**b**^ Data analysed is aggregated for more than one state

^**c**^ Empowered Action Group states include Bihar, Chhattisgarh, Jharkhand, Madhya Pradesh, Odisha, Rajasthan, Uttar Pradesh, and Uttarakhand

^**d**^ Data analysed based on a single state only

^**e**^ Data analysed at the district and sub-district levels (rural or urban)

^**f**^ Study conducted at the city/slum level, community level, village level

### Individual and interpersonal barriers

A total of 30 studies, including 17 quantitative [2, 4, 5, 9, 11, 18, 21, 22, 24, 29, 30, 36, 47–51], 10 qualitative [1, 8, 23, 26, 35, 52–56], and 3 mix-methods studies [25, 57, 58], reported that lack of knowledge and awareness are prominent barrier, resulting in incomplete or non-utilization of obstetric care services (OCS). Dalal et al. [2022] explained that due to a lack of awareness, women were unable to understand the processes and systems followed in the health facilities and felt uncomfortable visiting them, which directly influenced their utilization of OCS [35]. A study on currently breastfeeding women in Belagavi district, Karnataka, found that due to a lack of knowledge, most women were unable to address difficulties with breastfeeding on their own and depended on their mothers, mothers-in-law, or cousins [23]. A similar barrier was reported by many other studies conducted in other parts of India [19, 21, 24, 52].

Regarding ignorance towards utilization of OCS, Sarkar et al. [2021] explained that women who had a previous gestation and birth experience felt more confident about themselves and ignored the OCS services [8]. The statement of a 20-year-old woman who had registered her pregnancy well illustrates the ignorance towards utilization of antenatal care services:

*“I don’t like to go to the hospital. Although they took me to the hospital last time, it was not needed. I don’t go to the hospital for any disease. My sister gave me some tablets, but I did not take any of them. I don’t want any tablets.”* [58].

Lack of counselling on proper breastfeeding practice and their benefits were major barriers to delay in breastfeeding initiation or exclusive breastfeeding [20, 21, 49, 53]. Additionally, it was observed that mothers who devoted their maximum time to domestic chores did not have time to adhere to early breastfeeding [24]. A study in a hilly region of North India by Parashar et al. (2019) found a significant positive relationship between family support and outcome of pregnancy. For instance, inadequate support from family members yielded poor pregnancy outcomes in the form of low birth weight, preterm labour, low maternal weight gain, etc. [54].

It also emerged from our review that husbands opposed institutional delivery if their wives had experienced any complications during previous institutional delivery [28, 33]. Poor communication between health care providers and women availing OCS was another interpersonal barrier that led to poor utilization of OCS [5, 59, 60]. These types of barriers were primarily found in the forest and hill-dwelling communities as they are socially, culturally, and linguistically different from the mainstream population of India [34, 56].

### Social and cultural barriers

Belief in the efficacy of traditional herbs and healers [1, 4, 8, 33, 34, 55–57, 61–64] was a frequently reported barrier and was especially prevalent in rural Indian settings. Consequently, home delivery or using the services of a traditional birth attendant (TBA) who was receptive to the use of herbs was the preferred choice [1, 55]. Many studies revealed that the culture of discarding first milk (colostrum) was a major barrier to exclusive breastfeeding and newborn health [24, 27, 52, 60–62]. In this context, one traditional midwife from a rural community in Kalahandi district, Odisha, India explained her belief and practices about colostrum milk as follows:

*“Colostrum is the milk that has been in the breast for nine months and is impure through storage. It is therefore unhealthy for the baby and should be offered to mother earth for her blessings for the child.*” [60].

Several studies showed that pre-lacteal feeding rituals were major barriers to exclusive breastfeeding. The practice of giving newborn babies pre-lacteal feeds like jaggery, honey, sugar water, cow milk, and ghee was observed by many previous studies [27, 47, 49–51, 54].

Food restrictions during pregnancy and lactation were also identified as another common barrier and therefore a major concern for mother and child health [27, 34]. Most mothers believed that more food intake would cause digestive disorders in the baby. In an in-depth interview, a mother in the post-partum period from Palghar district in Maharashtra India, said:

*“In my postnatal period, up to 12 days after delivery, I ate less amount of food than the usual amount I eat because I believe that if I ate more, the baby would have had a bloated abdomen.”* [27].

In addition to the above beliefs and cultural practices, a wide range of taboos and superstitions were majorly reported obstacles in the utilization of OCS [11, 16, 18, 20, 26, 63]. For example, bathing the mother and child within 24 h of birth and applying mustard oil to the umbilical cord stump were highly risky practices observed in Bihar, India [62]. Delayed initiation of breastfeeding for several days until the pandit (Hindu priest) or maulana (Islam priest) blessed the infant [62], purification ceremonies [27, 53, 61, 62, 69], restriction of women’s mobility [27, 62, 69], and oil massages [27, 61, 53] were frequently reported barriers by various studies.

### Structural barriers

Poor economic status was a major hindrance in accessing OCS [1, 8, 9, 33, 47, 48, 51, 59, 63]. High cost of OCS services, such as cost of institutional delivery [15, 17, 25, 33, 35, 47, 59, 60, 66] and transportation cost [2, 14, 29, 60], were other important barriers in service utilization. These barriers were predominantly found among women who stood at the intersection of caste and poverty [25, 33, 35, 47]. The cost of referral services, including travel cost, was a major barrier that led to delay in reaching, or inability to reach, a referred health facility [1, 9, 33, 35]. The qualitative studies revealed that the cost of services was a key determining factor for the majority of women in deciding between home birth and institutional delivery [17, 35, 47]. A few studies found that due to the poor economic status of women and their families, they were unable to bear delivery cost and choose home delivery [1, 67, 55, 56, 60, 63, 64].

Studies consistently showed that due to financial constraints, women were not able to take local transport to visit a health facility and avail OCS [14, 60]. From a qualitative study, we found that despite free access to most maternal health care services and cash incentive schemes for pregnant women in India, most women still opted for home delivery. It because of the net value of the cash incentive was less than the total expense of facility-based childbirth in terms of both monetary and actual costs (healthcare expenditure, food, and transport expenses for the mother and her caregivers) [64]. Therefore, there was little motivation to opt for institutional delivery.

### Logistical barriers

Long distance to health facilities [1, 5, 9, 10, 12, 13, 15, 17, 28, 29, 33, 35, 47, 65, 50, 64] and lack of transportation facilities [11, 14, 16, 26, 28, 32, 33, 47, 48, 50, 58, 63] were major logistical barriers to accessing OCS. Such barriers were frequently reported from rural and geographically-isolated regions of India [10, 11, 35] and were a significant contributor to the underutilization of OCS services in the remote locations of India [10, 58, 60].

Poor road conditions [10, 14, 69] and lack or delay of ambulance service [10, 35, 55, 58, 64] were other major barriers in accessing OCS. A study conducted in Meghalaya explained the problems faced by women during their maternity period. According to that study, pregnant women faced a lot of difficulties while traveling to the district hospital during the rainy season. Due to the bad road conditions, the ambulance could not reach to them, forcing the women to get to the ambulance themselves after traversing muddy and rocky roads in rainy days [10]. Other studies reported that due to the long distance to health facilities, women were forced to deliver at home and depend on local herbs and practitioners [63, 60]. A study conducted in a rural region of Odisha found that about 50% of women were unable to visit a health facility for the ANC checkup and delivery due to the high cost of transportation facility [60]. A similar barrier was observed in the others states of India such as Karnataka [63], Kerala [58], and Meghalaya [69].

### Organizational barriers

Previous studies have documented that poor quality of treatment [4, 17, 25, 28, 33–35, 47, 50, 54, 64, 65, 68] and non-cooperative attitude of health professionals were responsible for hesitation in women and their families to seek OCS [14, 20, 25, 50, 54, 69]. Previous studies also reveal that disrespectful behavior of health professionals towards pregnant women is another major barrier to institutional delivery [20, 50, 54]. A pregnant woman who had delivered in a public hospital explained her experience as follows:

*“My first delivery was conducted in a government hospital. They admit you, allot you a bed, and then they treat you badly. The nurses scream so loudly as if they are Gods themselves. Rather than helping the women in labor, they abuse them. I was shouted at and even slapped in the labor room. I was so scared that I planned my second delivery at home. I received more care at home than at the hospital. I would advise others not to go there ever*” [54].

Women are also deterred from completing their ANC services due to health system-related barriers such as unavailability of trained health personnel [10, 36, 65, 68, 53, 54], long waiting time in hospitals [15, 26, 36, 50, 59, 65], inadequate equipment and shortage of drugs [9, 12, 26, 36, 47, 64, 68], and poor health infrastructure [9, 10, 15, 36, 68, 69]. In this context, a previous study explained that non-availability of adequate beds, labour/examination tables, and bed screens significantly reduced the volume of antenatal registrations and use of postnatal services [9, 12]. Similarly, a country-level study by Singh (2016) found that absence of electricity connection in a health facility was associated with an approximately 32% decrease in the volume of institutional deliveries and almost 10% decrease in ANC registrations [12]. Shortage of female doctors is also an important barrier that affects maternity care [26, 69]. A medical officer-in-charge from Uttar Pradesh made the following remarks during an in-depth interview:

*Doctor’s unavailability is a problem. Male doctors can’t do deliveries – women are shy and don’t let them do so. But the female staff is not enough in number and hence we are forced to get contractual staff* [67].

Other studies have also reported that the unavailability of labor rooms, long waiting time in health facilities, and lack of medical equipment and infrastructure drive women towards home delivery in India [12, 14, 26, 36, 65, 68]. All the above identified barriers are summarized in Table 3.

**Table 3:** Summary of barriers in OCS (n = 56)

Identified barriers	Qualitative Contributing Studies	N	Quantitative Contributing Studies	N	Mixed-Methods Contributing Studies	N	Total
**1. Individual Barriers**							
• Lack of knowledge/awareness/counselling	(1,8,23,26,35,57,59,60,64,65)	10	(2,4,5,9,11,18,21,22,24,28–30,47,50–53)	17	(25,67,68)	3	**30**
• Lack of social/family support	(26,35,60,62,66)	5	(11,13,15,16,28,33,51,53)	8	(25)	1	**14**
• Denial of permission by husband/family	(23,35)	2	(9,13,28,32,52)	5	(25,69)	2	**9**
• Ignorance/felt not necessary	(35,53,57)	3	(5,8,11,33,47)	5	(25)	1	**9**
• Lack of autonomy	(26,35,63,66)	4	(2,5)	2	(25)	1	**7**
• Working mother/resumption of work/domestic responsibility/lack of time	-	-	(13,19,20,33,50)	5	-	-	**5**
• Language or communication barrier	(62)	1	(5)	1	(69)	1	**3**
• Insecurity about visiting health facility/fear of medical intervention	(9)	1	(15)	1	-	-	**2**
• Family’s negative perception of OCS	(56)	1	(14)	1	-	-	**2**
**2. Cultural Barriers**							
• Belief in traditional herbs/healers	(1,8,34,58,63–66)	8	(4,33,54)	3	(67)	1	**12**
• Existing fear and taboos/misconceptions regarding medicine	(19,26,34,63)	4	(16,18)	2	(69)	1	**7**
• Pre-lacteal feeding rituals	(27,57–59)	4	(54)	1	(67)	1	**6**
• Discarding of colostrum	(23,27,57,58)	4	(54)	1	(69)	1	**6**
• Purification ceremony/birth rituals	(27,58,59,61)	4	(54)	1	-	-	**5**
• Social and cultural restrictions	(27,58)	2	(4,33)	2	-	-	**4**
• Food restrictions	(27,57)	2	(18,54)	2	-	-	**4**
**3. Structural Barriers**							
• Poor economic status	(1,62,63)	3	(8,9,33,47,49,52)	6	**-**	-	**9**
• High cost of service	(35,62)	2	(15,17,33,47,53)	5	(25,69)	2	**9**
• High cost of transportation	-	-	(2,14,29,69)	4			**4**
• Lack of money for transport	-	-	(14)	1	(69)	1	**2**
**4. Logistical Barriers**							
• Long distance to health facility	(1,35,48,66)	4	(5,9,12,13,15,17,28,29,33,47,51)	11	(10)	1	**16**
• Lack of transport facility	(56,63)	2	(11,14,16,26,28,32,33,47,49,51)	10	(68)	1	**13**
• Lack or delay of ambulance service	(35,64,66)	3	**-**	-	(10,68)	2	**5**
• Lack of money for transport	(63)	1	(2,29)	2	(68)	1	**4**
• Poor road conditions	(61)	1	(14)	1	(10)	1	**3**
• Geographically isolated area	(35)	1	(11)	1	(10)	1	**3**
• High cost of health service	(26)	1	(17)	1	**-**	-	**2**
**5. Organizational Barriers**							
• Poor quality of treatment	(26,34,35,48,56,60,66)	7	(4,17,28,33,47,51)	6	(25)	1	**14**
• Inadequate equipment and shortage of drugs	(26,56,66)	3	(9,12,36,47)	4	-	-	**7**
• Non-cooperative/abusive attitude of by health professionals/	(60,61)	2	(14,20,51)	3	(25)	1	**6**
• Lack of or poor health infrastructure	(56,61)	2	(9,15,36)	3	(10)	1	**6**
• Long waiting times/Facility not open	(26,48,62)	3	(15,36,51)	3	-	-	**6**
• Unavailability of trained health personnel	(56,59,60)	3	(36,48)	2	(10)	1	**6**
• Lack of ANM/ASHA support	(35)	1	(12,30,33)	3	-	-	**4**
• Inappropriate advice from health professionals	-	-	(4,22)	2	(25)	1	**3**
• Shortage of female doctors	(26,61)	2	-	-	-	-	**2**

**Note: ANM**: Auxiliary Nursing Midwifery; **ASHA**: Accredited Social Health Activist

## Discussion

The present systematic review evaluated the barriers in the utilization and provisioning of OCS using a mixed-methods approach. To our knowledge, this study is the first comprehensive review of barriers in OCS, ranging from ANC to PNC services. The present research categorized the barriers into five major themes, namely, individual and interpersonal, social and cultural, structural, logistical, and organizational barriers. This review found that lack of knowledge and awareness of, and counselling on, the importance of timely initiation of OCS as well as negative attitudes or ignorance towards OCS have frequently reported barriers and were largely observed among rural and marginalized women [5, 9, 11, 18, 35]. A systematic review conducted in the Indian context found similar individual and interpersonal barriers [38].

A few studies identified little or no involvement of the husband and lack of family support in obtaining OCS as important interpersonal barriers. A study revealed that many times, women with no additional barriers at the logistical or structural levels failed to complete OCS because of the unavailability of a suitable accompanying person [11–13, 15, 16, 35, 50, 67, 54]. In contrast, some studies highlighted that social and emotional support from the husband, family, or peer groups had the potential to influence OCS use [25, 57].

Another main finding of this review is that traditional beliefs and practices are still a strong barrier to utilizing OCS in India. Numerous negative social and cultural practices, such as dietary restrictions, belief in traditional herbs and healers, and misperceptions regarding colostrum, were frequently reported barriers during and after pregnancy. Women who belonged to rural and forest and hill-dwelling communities had more firm cultural beliefs than other women [60].

From a qualitative study, it was found that in rural settings, pregnant women dislike to reveal their pregnancy and try to maintain privacy and confidentiality; therefore, they refuse to avail of the facilities and prefer home delivery [69]. However, there is a scarcity of studies that identify the specific factors responsible for the prevalent cultural and traditional practices in Indian families.

According to the strategic approach to reproductive, maternal, newborn, child, and adolescent health (RMNCH + A) in India, inequitable distribution of OCS services, poor access to high-quality health care, and limited awareness among women are major barriers to the continuum of care [70]. Even though the government has made OCS more accessible at a low or no cost, the indirect cost of accessing health facilities is still a prominent barrier to utilizing the services. The costs related to transportation, medical expenses, ambulance service, and hospital stay and other indirect costs are higher than the free services provided by the Government of India. To remove the financial barriers and increase the utilization of OCS, the Government of India has successfully implemented various financial incentive schemes such as conditional cash transfer, voucher facilities, free comprehensive package of OCS facilities, etc. [71–73]. However, lack of trust in the health facilities and the perceived benefits are important factors for the incomplete utilization of OCS as mothers who have experienced poor treatment earlier are not allowed by their families to utilize OCS at health facilities again.

Long distance to health facilities are an important barrier preventing women from utilizing OCS. This review found a positive association between long distance to health facilities and increased chances of home delivery [10, 68, 69, 64]. Other important logistical barriers identified were difficulty reaching health facilities due to poor road networks, high transportation costs, and lack of ambulance services. Regarding service provisioning, weak infrastructure (lack of instruments, unavailability of obstetric drugs, lack of labour room), lack or shortage of health professionals, and long waiting hours are significant barriers to accessing OCS. Hilly regions suffer more shortage/ unavailability of essential obstetric care drugs and equipment [15]. A previous study mentioned that lack of essential obstetric drugs at a health facility might have a strong link to maternal mortality at the facility level.

This study found that poor communication between women and healthcare providers, disrespectful care (perceived or experienced), and discrimination based on caste and class are significant issues and provoke mothers to discontinue OCS [4].

Lasty, in the “BIMARU” states, logistical, cultural, and structural barriers such as poor health infrastructure [1, 35, 66, 67], long distance between labor rooms and resuscitation areas/poor access to public transport [1, 35], mistreatment during childbirth and lack of health facilitator [25, 67], traditional medical practices and rituals [1, 62], and poverty [1, 30, 35, 67] are more predominant barriers as compared to the non “BIMARU” states. In the empowered action group (EAG) states, poor knowledge regarding the importance of receiving OCS services and tendency not to receive the routine check-up treatments of ANC services are major barriers [3, 8].

### Strengths and limitations

This review explains the wide-ranging barriers to the provisioning and utilization of OCS, which can help public health professionals, policy-makers, and government and non-government organizations. The studies included in the review offer diverse perspectives across different geographical locations, cultures, and socioeconomic status, making our findings relevant across several cultural and socioeconomic groups. Since we included only those studies that qualified for the quality assessment based on the inclusion and exclusion criteria, we can be confident that the findings are reliable and acceptable.

Despite these strengths, there are some limitations as well. First, compared to the barriers to the utilization of OCS, very limited studies identified the barriers related to the provisioning of OCS. Second, most of the included studies were based on cross-sectional data; therefore, we could not find the differences in findings based on intervention. Third, in India, OCS barriers change according to rural and urban settings, but the authors were unable to find any studies that have examined the OCS in the rural and urban areas separately. Therefore, the current systematic review could not examine the difference in the barriers between the rural and the urban settings. Fourth, a few cross-sectional studies had a sample size of less than 100. While these studies may provide detailed descriptions of individual cases, it is difficult to make generalizations. Lastly, due to the differences in study designs, multiple tools were used to assess the quality of the included studies. Therefore, it’s difficult to compare quality across studies.

## Conclusion and recommendations

The present mixed-methods systematic review highlighted several important barriers, ranging from individual and cultural to structural, logistical, and organizational, which are prevalent in India. Some of the barriers, such as lack of knowledge, long distance to health facilities, lack of social/family support, and poor quality of treatment, need greater attention than the rest. This systematic review also observed that barriers to OCS are interdependent and influenced by various socioeconomic, cultural, psychological, and demographic factors. We found similar barriers to OCS in South Asia [53] and other developing countries [74]. Therefore, we suggest future studies to examine the social and demographical determinants of OCS and explore the strategies, focusing particularly on lower- and middle-income countries.

At the India level, to increase the utilization and provisioning of OCS, the Government of India needs to develop strategies at the individual as well as organizational levels. In the long term, these steps will reduce the identified barriers. Lastly, innovative interventions and program implementation at the community and village levels could be a contributory step towards improving the utilization of OCS in India.

### Electronic supplementary material

Below is the link to the electronic supplementary material.

**Supplementary Material 1: Additional File 1.** PRISMA 2009 Checklist

**Supplementary Material 2: Additional File 2.** Results of an electronic search identified through different databases and using keywords

**Supplementary Material 3: Additional File 3.** Sample data extraction forms

**Supplementary Material 4: Additional File 4.** Quality assessment tool for included articles

## Data Availability

All data generated or analyzed during this study are included in this published article (and its supplementary information files).
