# Supplementary material for: Barriers in utilization and provisioning of obstetric care services (OCS) in India: a mixed-methods systematic review
Source: BMC Pregnancy Childbirth. 2024 Jan 2;24:16. doi: 10.1186/s12884-023-06189-x (PMC10759396; doi:10.1186/s12884-023-06189-x)
Supplement: Supplementary file 2 — Supplementary Material 2: Additional File 2. Results of an electronic search identified through different databases and using keywords [file 12884_2023_6189_MOESM2_ESM.docx]

**Additional File 2. Results of an electronic search identified through different databases and using keywords**

1. **PubMed**

| **Keywords search** | **Date of search** | **Search engine used** | **Number of publications retrieved** |
| --- | --- | --- | --- |
| (((("obstetric"[All Fields] OR "obstetrically"[All Fields] OR "obstetrics"[MeSH Terms] OR "obstetrics"[All Fields] OR "obstetrical"[All Fields]) AND "care"[All Fields] AND ("service"[All Fields] OR "service s"[All Fields] OR "serviced"[All Fields] OR "services"[All Fields] OR "services s"[All Fields] OR "servicing"[All Fields])) OR ("maternal health"[MeSH Terms] OR ("maternal"[All Fields] AND "health"[All Fields]) OR "maternal health"[All Fields])) AND 2000/01/01:2022/06/30[Date - Publication] AND (("barrier"[All Fields] OR "barrier s"[All Fields] OR "barriers"[All Fields] OR ("obstacle"[All Fields] OR "obstacles"[All Fields])) AND 2000/01/01:2022/06/30[Date - Publication]) AND (("India"[MeSH Terms] OR "India"[All Fields] OR "India s"[All Fields] OR "India’s"[All Fields] OR (("Indian"[All Fields] OR "Indian s"[All Fields] OR "Indians"[All Fields]) AND ("state"[All Fields] OR "state’s"[All Fields] OR "stated"[All Fields] OR "states"[All Fields] OR "stating"[All Fields]))) AND 2000/01/01:2022/06/30[Date - Publication])) AND (2000/1/1:2022/6/30[pdat])  ((((maternal health) OR (maternal health services)) OR (obstetric care service)) AND (barriers)) AND (India) AND (2000/1/1:2022/6/30[pdat]) | **April – May, 2022** | **PubMed** | **1153** |

1. **SCOPUS**

| **Keywords search** | **Date of search** | **Search engine used** | **Number of publications retrieved** |
| --- | --- | --- | --- |
| ( TITLE-ABS-KEY ( maternal AND health ) OR TITLE-ABS-KEY ( obstetric AND care AND service ) OR TITLE-ABS-KEY ( challenges OR disparity ) ) AND PUBYEAR > 2009 AND PUBYEAR < 2023 AND ( LIMIT-TO ( AFFILCOUNTRY , "India" ) ) AND ( LIMIT-TO ( LANGUAGE , "English" ) )  ( TITLE-ABS-KEY ( maternal  AND health )  OR  TITLE-ABS-KEY ( maternal  AND health  AND care )  OR  TITLE-ABS-KEY ( barriers  OR  obstacles )  AND  AFFILCOUNTRY ( india ) )  AND  PUBYEAR  >  2009  AND  PUBYEAR  <  2023  AND  ( LIMIT-TO ( LANGUAGE ,  "English" ) )  ( TITLE-ABS-KEY ( maternal  AND health )  OR  TITLE-ABS-KEY ( obstetric  AND care  AND service  AND combination )  AND  TITLE-ABS-KEY ( barriers  OR  obstacles ) )  AND  PUBYEAR  >  2009  AND  PUBYEAR  <  2023  AND  ( LIMIT-TO ( AFFILCOUNTRY ,  "India" ) )  AND  ( LIMIT-TO ( LANGUAGE ,  "English" ) )  ( TITLE-ABS-KEY ( maternal  AND health )  OR  TITLE-ABS-KEY ( maternal  AND health  AND care )  AND  TITLE-ABS-KEY ( barriers  OR  obstacles ) )  AND  PUBYEAR  >  2009  AND  PUBYEAR  <  2023  AND  ( LIMIT-TO ( AFFILCOUNTRY ,  "India" ) )  AND  ( LIMIT-TO ( LANGUAGE ,  "English" ) )  ( TITLE-ABS-KEY ( maternal  AND health )  OR  TITLE-ABS-KEY ( obstetric  AND care  AND service  AND combination )  AND  TITLE-ABS-KEY ( indian  AND states ) )  AND  PUBYEAR  >  2009  AND  PUBYEAR  <  2023  AND  ( LIMIT-TO ( AFFILCOUNTRY ,  "India" ) )  AND  ( LIMIT-TO ( LANGUAGE ,  "English" ) )  ( TITLE-ABS-KEY ( maternal AND health ) OR TITLE-ABS-KEY ( maternal AND health AND care ) OR TITLE-ABS-KEY ( obstetric AND care AND service AND combination ) AND AFFILCOUNTRY ( barriers OR obstacles ) OR TITLE-ABS-KEY ( challenges OR disparity AND combination ) ) AND PUBYEAR > 2009 AND PUBYEAR < 2023 AND ( LIMIT-TO ( AFFILCOUNTRY , "India" ) ) AND ( LIMIT-TO ( LANGUAGE , "English" ) ) | **June, 2022** | **SCOPUS** | **716** |

1. **Web of Science**

| # | Search Query | Date of search | Search engine used | Number of publications retrieved |
| --- | --- | --- | --- | --- |
| 1 | (ts= (Maternal health* near/3 obstetric care services* OR Maternal health services*)) Timespan: 2000-01-01 to 2022-06-30 | **May, 2022** | **Web of Science** | **463** |
| 2 | (ts= ('utilization of obstetric care services* OR provisioning of obstetric care services*)) Timespan: 2000-01-01 to 2022-06-30 |  |  |  |
| 3 | (((TI=(provisioning of obstetric care services*))) OR TI=(utilization obstetric care services)) Timespan: 2000-01-01 to 2022-06-30 |  |  |  |
| 4 | ((TS=(maternal health India)) OR TS=(maternal services India)) OR TS=(Barrier in maternal health) Timespan: 2000-01-01 to 2022-06-30 |  |  |  |
| 5 | (((TS=(antenatal care service*)) OR TS=(postnatal service*)) OR TS=(postpartum care*)) AND TS=(perinatal care*) Timespan: 2000-01-01 to 2022-06-30 |  |  |  |
| 6 | (TS=(Barrier in utilization of obstetric care service*)) OR TS=(Challenges in utilization of obstetric care service*) Timespan: 2000-01-01 to 2022-06-30 |  |  |  |
| 7 | (TI=(Barrier in utilization of obstetric care service)) OR TI=(Barrier in maternal health service) Timespan: 2000-01-01 to 2022-06-30 |  |  |  |
| 8 | Barrier in utilization of obstetric care service* (Topic) |  |  |  |
| 9 | Barrier in utilization of obstetric care service* (Topic) and INDIA (Countries/Regions) |  |  |  |
| 10 | ((TS=(Barrier in obstetric care service in India)) OR TS=(Barrier in maternal care service in India*)) OR TS=(Barrier in emergency obstetric care service in India*)  Timespan: 2000-01-01 to 2022-06-30 |  |  |  |
| 1 | #10 OR #9 OR #8 OR #7 OR #6 OR #5 OR #4 OR #3 OR #2 OR #1 Timespan: 2000-01-01 to 2022-06-30 |  |  |  |

1. **Google Scholar**

| **Keywords search** | **Date of search** | **Search engine used** | **Number of publications retrieved** |
| --- | --- | --- | --- |
| "Obstetric care service", OR "Emergency Obstetric care service", OR "Maternal Health", OR "Maternal health care" AND "barriers", OR "obstacles", OR "challenges AND (“India” OR “Indian States”) " (LIMIT-TO (LANGUAGE, “English)) AND (LIMIT-TO (pub-date IS 20100101 BEF 20220630)) | **June, 2022** | **Google Scholar** | **369** |

| **Keywords search** | **Date of search** | **Search engine used** | **Number of publications retrieved** |
| --- | --- | --- | --- |
| "Obstetric care service", OR "Emergency Obstetric care service", OR "Maternal Health", OR "Maternal health care" AND "barriers", OR "obstacles", OR "challenges AND (“India” OR “Indian States”) " (LIMIT-TO (LANGUAGE, “English)) AND (LIMIT-TO (pub-date IS 20100101 BEF 20220630))  **Science Direct Search guide:**  <https://dev.elsevier.com/tips/ScienceDirectSearchTips.htm> | **April, 2022** | **Science Direct** | **216** |

1. **Science Direct**
2. **All the Keywords related to Obstetric care service [MeSH terms]:**

‘Obstetric care service’; OR ‘Emergency Obstetric care service’; OR ‘utilization of obstetric care services;’ OR provisioning of Obstetric care service’ OR ‘obstetric complication’ OR ‘emergency obstetric care’ OR ‘Maternal Health’; OR ‘Maternal health care’; OR ‘Maternity care’ OR ‘pregnancy complication’, OR ‘antenatal care service’ OR ‘postnatal service’, OR ‘postpartum care’ OR ‘perinatal care’ OR ‘child delivery’, OR ‘institutional delivery’, OR ‘home delivery’ OR ‘breastfeeding’.

**Combination with**

Combination of (**AND**) ‘barriers’, OR ‘obstacles’, OR ‘challenges’, OR ‘social inequality’ OR ‘social disparity’ OR ‘factors’ OR ‘beliefs’ OR ‘prevalence’, OR ‘determinants’, ‘predictors, OR ‘associated OR ‘hinder’ OR ‘hindrance’ OR disparity combination with ‘India’ OR ‘Indian states’ OR ‘Indian cities’.

**Individual and interpersonal barriers:** ‘Knowledge’, ‘awareness’, ‘ignorance’, ‘counselling’ ‘social support’, ‘family support’, ‘social support’, ‘husband support’, ‘poor communication’.

**Social and cultural barriers: ‘**Cultural practices’, ‘taboos’, ‘custom’, ‘rituals’, ‘traditional herbs’, ‘food restriction’, ‘superstitions’, ‘traditional practice’, ‘stigma’, ‘misconception’.

**Structural barriers: ‘**Poverty’, ‘economic status’, ‘cost of service’, ‘out of pocket expenditure’, ‘income’, ‘caste’.

**Logistical barriers:** ‘Transportation’, ‘distance’, ‘road condition’, ‘ambulance service’, ‘geographical isolation’.

**Organization barriers: ‘**Attitude’, ‘disrespectful behaviours’, ‘long waiting time’, ‘shortage of drugs’, ‘shortage of equipment’, ‘health infrastructure’.
