# Supplementary material for: Barriers in utilization and provisioning of obstetric care services (OCS) in India: a mixed-methods systematic review
Source: BMC Pregnancy Childbirth. 2024 Jan 2;24:16. doi: 10.1186/s12884-023-06189-x (PMC10759396; doi:10.1186/s12884-023-06189-x)
Supplement: Supplementary file 3 — Supplementary Material 3: Additional File 3. Sample data extraction forms [file 12884_2023_6189_MOESM3_ESM.docx]

**Additional File 3. Sample data extraction forms**

**A. Sample data extraction forms for quantitative studies**

| **Data extraction** | **Response** | **Reviewers comment** | **Support for judgement** |
| --- | --- | --- | --- |
| Study ID |  |  |  |
| Author Name and Publication Year |  |  |  |
| Study Design |  |  |  |
| Objective |  |  |  |
| Geographic setting |  |  |  |
| Study methods |  |  |  |
| Study focused areas |  |  |  |
| study year |  |  |  |
| Sample size |  |  |  |
| Method of data collection |  |  |  |
| Data source |  |  |  |
| Results of Quality Assessment |  |  |  |
| Inclusion Criteria |  |  |  |
| Exclusion Criteria |  |  |  |
| Data Collection Instruments |  |  |  |
| Response Rate / Outcome Data |  |  |  |
| Results |  |  |  |
| Overall quality |  |  |  |

**B. Barrier extraction form (Quantitative, qualitative and Mixed-methods studies)**

| **Study ID** |  | | |
| --- | --- | --- | --- |
| **Author Name and Publication Year** |  | | |
| **Barrier theme** | **Quantitative** | **Qualitative** | **Mixed-methods studies** |
| Individual and interpersonal barriers | #Prepare the list of barriers: | #Prepare the list of barriers: | #Prepare the list of barriers: |
| Social and cultural barriers | #Prepare the list of barriers: | #Prepare the list of barriers: | #Prepare the list of barriers: |
| Structural barriers | #Prepare the list of barriers: | #Prepare the list of barriers: | #Prepare the list of barriers: |
| Logistical barriers | #Prepare the list of barriers: | #Prepare the list of barriers: | #Prepare the list of barriers: |
| Organizational barriers | #Prepare the list of barriers: | #Prepare the list of barriers: | #Prepare the list of barriers: |

**C. Sample data extraction forms for qualitative studies**

| **Data extraction** | **Response** | **Reviewers comment** | **Support for judgement** |
| --- | --- | --- | --- |
| Study ID |  |  |  |
| Author Name and Publication Year |  |  |  |
| Study Design |  |  |  |
| Objective |  |  |  |
| Geographic setting |  |  |  |
| Study methods |  |  |  |
| study year |  |  |  |
| Sample size |  |  |  |
| Method of data collection |  |  |  |
| Results of Quality Assessment |  |  |  |
| Overall quality |  |  |  |
| **Qualitative Themes** |  |  |  |
| Qualitative Data | *[Insert selected qualitative quotation from ‘findings’ or ‘results’ sections]* | | |
