# Supplementary material for: Barriers in utilization and provisioning of obstetric care services (OCS) in India: a mixed-methods systematic review
Source: BMC Pregnancy Childbirth. 2024 Jan 2;24:16. doi: 10.1186/s12884-023-06189-x (PMC10759396; doi:10.1186/s12884-023-06189-x)
Supplement: Supplementary file 4 — Supplementary Material 4: Additional File 4. Quality assessment tool for included articles [file 12884_2023_6189_MOESM4_ESM.docx]

**Additional File 4. Quality assessment tool for included articles**

| **Sr. No.** | **Author(s)** | **Year** | **Statement** | **Methodology** | **Design** | **Recruitment strategy** | **Data collection** | **Relationship between R & and P** | **Ethical issues** | **Data analysis** | **Findings** | **Research value** | **Total Score** | **Quality of Study** |
| --- | --- | --- | --- | --- | --- | --- | --- | --- | --- | --- | --- | --- | --- | --- |
|  |  |  | **Q 1** | **Q 2** | **Q 3** | **Q 4** | **Q 5** | **Q 6** | **Q 7** | **Q 8** | **Q 9** | **Q 10** |  |  |
| 1 | Jat et al. [56] | 2015 |  |  |  |  |  |  |  |  |  |  | **9** | **Good** |
| 2 | Majra & Silan [59] | 2016 |  |  |  |  |  |  |  |  |  |  | **6** | **Low** |
| 3 | Vidler et al. [63] | 2016 |  |  |  |  |  |  |  |  |  |  | **9.5** | **Good** |
| 4 | Vincent [64] | 2017 |  |  |  |  |  |  |  |  |  |  | **6** | **Low** |
| 5 | Vellakkal et al. [66] | 2017 |  |  |  |  |  |  |  |  |  |  | **9** | **Good** |
| 6 | Contractor et al. [34] | 2018 |  |  |  |  |  |  |  |  |  |  | **10** | **Good** |
| 7 | Bhattacharyya et al. [55] | 2018 |  |  |  |  |  |  |  |  |  |  | **9** | **Good** |
| 8 | Dehury et al. [27] | 2018 |  |  |  |  |  |  |  |  |  |  | **7.5** | **Moderate** |
| 9 | Sarkar et al. [61] | 2018 |  |  |  |  |  |  |  |  |  |  | **6** | **Low** |
| 10 | Vail et al. [1] | 2018 |  |  |  |  |  |  |  |  |  |  | **9** | **Good** |
| 11 | Parashar et al. [60] | 2019 |  |  |  |  |  |  |  |  |  |  | **6.5** | **Low** |
| 12 | Kamble et al.[57] | 2020 |  |  |  |  |  |  |  |  |  |  | **6** | **Low** |
| 13 | Legare et al. [58] | 2020 |  |  |  |  |  |  |  |  |  |  | **7.5** | **Moderate** |
| 14 | Charantimath et al. (23) | 2020 |  |  |  |  |  |  |  |  |  |  | **10** | **Good** |
| 15 | Singh [62] | 2020 |  |  |  |  |  |  |  |  |  |  | **6** | **Low** |
| 16 | Zahoor et al.[65] | 2020 |  |  |  |  |  |  |  |  |  |  | **6** | **Low** |
| 17 | Athavale et al. [26] | 2020 |  |  |  |  |  |  |  |  |  |  | **10** | **Good** |
| 18 | Dalal et al.[35] | 2022 |  |  |  |  |  |  |  |  |  |  | **10** | **Good** |

1. **Quality assessment of qualitative studies (n=18)**

**Note: Good (9-10 score), Moderate (7.5-8.5 score), Low (6.0 – 7.0 score)**

| **Category** | **Yes** | **Partial** | **No** |
| --- | --- | --- | --- |
| **Score** | **1** | **0.5** | **0** |
| **Colour coded** |  |  |  |

1. **Quality assessment of quantitative studies (n=33)**

| **Sr. No.** | **Author(s)** | **Year** | **1** | **2** | **3** | **4** | **5** | **6** | **7** | **8** | **9** | **10** | **11** | **12** | **13** | **14** | **Total Score** | **Quality of Study** |
| --- | --- | --- | --- | --- | --- | --- | --- | --- | --- | --- | --- | --- | --- | --- | --- | --- | --- | --- |
| 1 | Kesterton et al.[17] | 2010 |  |  |  |  |  |  |  |  |  |  |  |  |  |  | **10** | **Moderate** |
| 2 | Chimankar and Sahoo [32] | 2011 |  |  |  |  |  |  |  |  |  |  |  |  |  |  | **8** | **Moderate** |
| 3 | Mumbare & Rege [4] | 2011 |  |  |  |  |  |  |  |  |  |  |  |  |  |  | **9** | **Moderate** |
| 4 | Ranganath and Poornima [52] | 2011 |  |  |  |  |  |  |  |  |  |  |  |  |  |  | **7** | **Moderate** |
| 5 | Pahwa and sood [51] | 2013 |  |  |  |  |  |  |  |  |  |  |  |  |  |  | **7** | **Moderate** |
| 6 | Bhattacherjee et al [33] | 2013 |  |  |  |  |  |  |  |  |  |  |  |  |  |  | **9** | **Moderate** |
| 7 | Sharma et al [30] | 2014 |  |  |  |  |  |  |  |  |  |  |  |  |  |  | **11** | **Good** |
| 8 | Kumar and Dansereau [36] | 2014 |  |  |  |  |  |  |  |  |  |  |  |  |  |  | **11** | **Good** |
| 9 | Joshi et al. [18] | 2014 |  |  |  |  |  |  |  |  |  |  |  |  |  |  | **8** | **Moderate** |
| 10 | Paudel [29] | 2014 |  |  |  |  |  |  |  |  |  |  |  |  |  |  | **7** | **Moderate** |
| 11 | Bhimani et al. [9] | 2016 |  |  |  |  |  |  |  |  |  |  |  |  |  |  | **7** | **Moderate** |
| 12 | Singh, 2016 [12] | 2016 |  |  |  |  |  |  |  |  |  |  |  |  |  |  | **11** | **Good** |
| 13 | Kakati et al. [11] | 2016 |  |  |  |  |  |  |  |  |  |  |  |  |  |  | **8** | **Moderate** |
| 14 | Apum and Nochi, [47] | 2017 |  |  |  |  |  |  |  |  |  |  |  |  |  |  | **7** | **Moderate** |
| 15 | Udgiri [54] | 2018 |  |  |  |  |  |  |  |  |  |  |  |  |  |  | **7** | **Moderate** |
| 16 | Fulpagare et al. [5] | 2019 |  |  |  |  |  |  |  |  |  |  |  |  |  |  | **10** | **Moderate** |
| 17 | Bhanderi et al. [21] | 2019 |  |  |  |  |  |  |  |  |  |  |  |  |  |  | **6** | **Moderate** |
| 18 | Randhawa et al. [20] | 2019 |  |  |  |  |  |  |  |  |  |  |  |  |  |  | **10** | **Moderate** |
| 19 | Agarwal et al. [19] | 2019 |  |  |  |  |  |  |  |  |  |  |  |  |  |  | **9** | **Moderate** |
| 20 | Narzary et al. [28] | 2019 |  |  |  |  |  |  |  |  |  |  |  |  |  |  | **9** | **Moderate** |
| 21 | Singh et al. [53] | 2019 |  |  |  |  |  |  |  |  |  |  |  |  |  |  | **9** | **Moderate** |
| 22 | Juyal et al.[49] | 2020 |  |  |  |  |  |  |  |  |  |  |  |  |  |  | **8** | **Moderate** |

**Continued.**

| **Sr. No.** | **Author(s)** |  | **1** | **2** | **3** | **4** | **5** | **6** | **7** | **8** | **9** | **10** | **11** | **12** | **13** | **14** | **Total Score** | **Quality of Study** |
| --- | --- | --- | --- | --- | --- | --- | --- | --- | --- | --- | --- | --- | --- | --- | --- | --- | --- | --- |
| 23 | Ogbo et al. [2] | 2019 |  |  |  |  |  |  |  |  |  |  |  |  |  |  | **11** | **Good** |
| 24 | Pariya and Das [22] | 2020 |  |  |  |  |  |  |  |  |  |  |  |  |  |  | **6** | **Moderate** |
| 25 | Nair et al. [50] | 2020 |  |  |  |  |  |  |  |  |  |  |  |  |  |  | **9** | **Moderate** |
| 26 | Verma et al. [13] | 2020 |  |  |  |  |  |  |  |  |  |  |  |  |  |  | **7** | **Moderate** |
| 27 | Marwein and Rao [14] | 2020 |  |  |  |  |  |  |  |  |  |  |  |  |  |  | **10** | **Moderate** |
| 28 | Purohit, [15] | 2021 |  |  |  |  |  |  |  |  |  |  |  |  |  |  | **8** | **Moderate** |
| 29 | Gupta et al. [48] | 2021 |  |  |  |  |  |  |  |  |  |  |  |  |  |  | **10** | **Moderate** |
| 30 | Venkatachalapathi [24] | 2021 |  |  |  |  |  |  |  |  |  |  |  |  |  |  | **7** | **Moderate** |
| 31 | Sarkar et al. [8] | 2021 |  |  |  |  |  |  |  |  |  |  |  |  |  |  | **10** | **Moderate** |
| 32 | Zacharias et al. [16] | 2021 |  |  |  |  |  |  |  |  |  |  |  |  |  |  | **7** | **Moderate** |
| 33 | Zaveri et al. [3] | 2022 |  |  |  |  |  |  |  |  |  |  |  |  |  |  | **11** | **Good** |

**Note:** Quality was rated as poor (0–4 score out of 14 questions), rated as moderate (5–10 score out of 14 questions), rated as good (11–14 score out of 14 questions); NA: not applicable, NR: not reported.

| **Category** | **Yes** | **No** | **CD/NA/NR** |
| --- | --- | --- | --- |
| **Score** | **1** | **0** | **-** |
| **Colour coded** |  |  |  |

**Quantitative quality assessment questions**

1. Was the research question or objective in this paper clearly stated?
2. Was the study population clearly specified and defined?
3. Was the participation rate of eligible persons at least 50%?
4. Were all the subjects selected or recruited from the same or similar populations (including the same time period)? Were inclusion and exclusion criteria for being in the study prespecified and applied uniformly to all participants?
5. Was a sample size justification, power description, or variance and effect estimates provided?
6. For the analyses in this paper, were the exposure(s) of interest measured prior to the outcome(s) being measured?
7. Was the timeframe sufficient so that one could reasonably expect to see an association between exposure and outcome if it existed?
8. For exposures that can vary in amount or level, did the study examine different levels of the exposure as related to the outcome (e.g., categories of exposure, or exposure measured as continuous variable)?
9. Were the exposure measures (independent variables) clearly defined, valid, reliable, and implemented consistently across all study participants?
10. Was the exposure(s) assessed more than once over time?
11. Were the outcome measures (dependent variables) clearly defined, valid, reliable, and implemented consistently across all study participants?
12. Were the outcome assessors blinded to the exposure status of participants?
13. Was loss to follow-up after baseline 20% or less?
14. Were key potential confounding variables measured and adjusted statistically for their impact on the relationship between exposure(s) and outcome(s)?

| **Sr. No.** | **Author(s)** | **Year** | **1** | **2** | **3** | **4** | **5** | **Score** | **Quality of Study** |
| --- | --- | --- | --- | --- | --- | --- | --- | --- | --- |
| **1** | Debnath et al. [67] | 2021 |  |  |  |  |  | **5** | **Good** |
| **2** | Jose et al. [68] | 2014 |  |  |  |  |  | **4** | **Good** |
| **3** | Sahoo et al. [69] | 2017 |  |  |  |  |  | **4** | **Good** |
| **4** | Sudhinaraset et al. [25] | 2016 |  |  |  |  |  | **5** | **Good** |
| **5** | Momin and Dutta [10] | 2021 |  |  |  |  |  | **3** | **Moderate** |

1. **Quality assessment of mix-method studies (n=5)**

**Note:** Quality was rated as poor (0–1 score out of 5 questions), rated as moderate (2–3 score out of 5 questions), rated as good (4–5 score out of 5 questions)

| **Category** | **Yes** | **No** |
| --- | --- | --- |
| **Score** | **1** | **0** |
| **Colour coded** |  |  |

**Mixed method quality assessment questions**

1. Is there an adequate rationale for using a mixed methods design to address the research question?
2. Are the different components of the study effectively integrated to answer the research question?
3. Are the outputs of the integration of qualitative and quantitative components adequately interpreted?
4. Are divergences and inconsistencies between quantitative and qualitative results adequately addressed?
5. Do the different components of the study adhere to the quality criteria of each tradition of the methods involved?
